# Stem Cell-Based Tissue Engineering for the Treatment of Burn Wounds: A Systematic Review of Preclinical Studies

**DOI:** 10.1007/s12015-022-10341-z

**Published:** 2022-02-12

**Authors:** Alissa Olga Lukomskyj, Nikitha Rao, Lei Yan, Jasmine Sarah Pye, Haiyan Li, Bin Wang, Jiao Jiao Li

**Affiliations:** 1 Kolling Institute, Faculty of Medicine and Health, University of Sydney, St Leonards, NSW 2065 Australia; 2 School of Biomedical Engineering, Faculty of Engineering and IT, University of Technology Sydney, Sydney, NSW 2007 Australia; 3 Department of Orthopedics, Shanxi Medical University Second Affiliated Hospital, Taiyuan, 030001 China; 4 Chemical and Environmental Engineering, School of Engineering, RMIT University, Melbourne, VIC 3000 Australia; 5 Department of Orthopaedic Surgery, The First Affiliated Hospital, Zhejiang University School of Medicine, Hangzhou, 315000 China

**Keywords:** Tissue engineering, Stem cells, Biomaterials, Burn wounds, Animal models

## Abstract

Burn wounds are a devastating type of skin injury leading to severe impacts on both patients and the healthcare system. Current treatment methods are far from ideal, driving the need for tissue engineered solutions. Among various approaches, stem cell-based strategies are promising candidates for improving the treatment of burn wounds. A thorough search of the Embase, Medline, Scopus, and Web of Science databases was conducted to retrieve original research studies on stem cell-based tissue engineering treatments tested in preclinical models of burn wounds, published between January 2009 and June 2021. Of the 347 articles retrieved from the initial database search, 33 were eligible for inclusion in this review. The majority of studies used murine models with a xenogeneic graft, while a few used the porcine model. Thermal burn was the most commonly induced injury type, followed by surgical wound, and less commonly radiation burn. Most studies applied stem cell treatment immediately post-burn, with final endpoints ranging from 7 to 90 days. Mesenchymal stromal cells (MSCs) were the most common stem cell type used in the included studies. Stem cells from a variety of sources were used, most commonly from adipose tissue, bone marrow or umbilical cord, in conjunction with an extensive range of biomaterial scaffolds to treat the skin wounds. Overall, the studies showed favourable results of skin wound repair in animal models when stem cell-based tissue engineering treatments were applied, suggesting that such strategies hold promise as an improved therapy for burn wounds.

**Graphical abstract:**

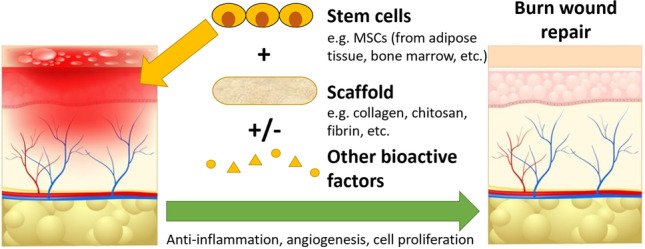

**Supplementary Information:**

The online version contains supplementary material available at 10.1007/s12015-022-10341-z.

## Introduction

Skin is the body’s largest organ and the first line of defence against injury or infection [1]. Burn wounds are a primary cause of skin damage, which have significant negative impacts on the physical and mental health of patients, leading to reduced quality of life. Burns are considered a global health issue and account for 180,000 deaths annually [2]. In Australia alone, there were 5,430 hospitalisations for burns in 2013–14, constituting 1.2% of all injury-related cases during this time period [3]. The average cost of burns treatment for Australian patients is a staggering $71,056 [3], which may be increased by several fold depending on the severity of the burn. High threat to life cases alone prolong the standard 7 days of hospitalisation to 17 days, evidencing the huge socio-economic burden of burn wounds on both the patient and healthcare facilities [3]. Furthermore, burn wounds lead to poorer quality of life for the patient [4], as they cause a decline in physical functioning and may impact the capacity to work, body image, basic abilities and mental health of patients [5]. The loss of 15% of the total body surface area (TBSA) from burn injury is sufficient to be considered life-threatening [6, 7]. Burn wounds are also more complex to manage and treat when they occur in paediatric patients under 5 years, or elderly persons over 60 years of age [7].

The complex structure and function of skin pose numerous challenges in regeneration after burn injury. The skin is composed of three distinct layers: the epidermis, dermis and hypodermis. The epidermis acts as the primary defence layer against organic elements and offers protection from the external environment. Thickness varies depending on the region of the body. The dermis is the second major layer of skin and is composed of collagen, elastin, electrolytes, and water. It also varies in thickness depending on location, ranging from 200 μm in the eyelids to 3 mm in the back. The hypodermis provides insulation from cold and violent trauma, and also acts to store energy [8]. Epidermal appendages consist of hair follicles, sebaceous glands, apocrine glands and eccrine glands [9]. Hair follicles are distributed all over the body except for the palms and soles, providing protection from ultraviolet radiation and preventing foreign material from entering the body. Sebaceous glands are found where hair is present and are responsible for sebum production and secretion to lubricate the skin. Apocrine glands are only found in certain areas of the body and their odourless secretion reacts with bacteria on the skin’s surface to produce body odour. Eccrine glands are present all over the skin’s surface and aid in retaining moisture and regulating body temperature, through the controlled release of sweat. In burn wounds, the inflicted damage leads to the death of skin cells and leaves the area susceptible to infection. Effective treatments for burn injury must first overcome the huge barrier of vascularisation, as failure to provide an adequate blood supply to the skin can result in necrosis, infection, sloughing or sepsis [6]. Epidermal appendages lost at the site of damage also typically fail to regenerate, leaving behind tissue scars from the poorly reconstituted collagen matrix [10].

Current clinical practices for treating burn wounds include skin grafting, skin substitutes, and negative pressure wound therapy (NPWT). The standard treatment for burn wounds involves early excision of necrotic tissue followed by autologous skin grafting procedures [11]. However, the limited availability of autologous skin becomes an issue for patients with severe burn injuries [12]. This problem can be solved by repeated harvesting of the donor site, but the site must re-epithelialise within 2 to 3 weeks to reduce scarring [13]. Biological skin substitutes including amnion and cultured epithelial autografts (CEA) have proven effective in the treatment of burn wounds, although the presence of allogeneic products presents a risk of contamination and disease transmission [14]. Synthetic skin substitutes such as Biobrane® and Suprathel® have shown favourable results in accelerating healing time and reducing pain, but still pose risks of infection and hypertrophic scarring [15]. For NPWT, a clinical trial involving small-area, thermal paediatric burns showed that this expedited re-epithelialisation [16]. However, NPWT can be problematic for both patients and caregivers due to the physical burden, technical difficulties and mechanical issues. Hence, current clinical approaches are associated with numerous practical drawbacks for the patient, and also lack efficacy in the complete renewal of skin that has been compromised by burn wounds. These limitations call for the need to develop tissue engineered treatment methods.

Tissue engineering approaches have become a significant area of interest for the treatment of burn wounds. Tissue engineered skin replacements have great potential for widespread applications in the field of wound healing, particularly to address the limited availability of autologous skin [17]. Recent advances include the exploration of strategies involving stem cells, biomaterials, and advanced manufacturing methods such as 3D printing to produce effective, alternative treatments. For instance, the role of adipose tissue-derived stem cell (AdSC) transplantation in skin repair has been demonstrated in a murine model [18], where enhanced tissue regeneration was evidenced by increased cell proliferation, a higher degree of neovascularisation, and up-regulation of the epidermal growth factor (EGF) protein. Moreover, a variety of biomaterials have been investigated as scaffolds to support skin regeneration, such as silk fibroin [19], metal-doped calcium silicate [20], and polymeric hydrogel scaffolds [21]. These biomaterials have been shown to favour re-epithelialisation and angiogenesis, reduce the risk of post-injury infection, and possess excellent biocompatibility. Additionally, new methods have been developed to ‘print’ functional living skin, such as by using a biomimetic bio-ink and digital light processing-based 3D printing technology [22]. This approach was shown to promote efficient neovascularisation by mimicking the structure of natural skin, which induced dermal regeneration in a large animal model. Furthermore, Integra [23], a widely recognised dermal replacement technology worldwide, provides a scaffold for endogenous cell ingrowth and dermal stroma synthesis following healing. Integra has been used clinically as a skin substitute [24], and shown to reduce wound surface area and accelerate healing [25]. However, a major challenge lies in its susceptibility to infection, caused by the collection of haematomas and seromas beneath the artificial skin substitute [26].

Currently, stem cells used for skin tissue engineering are in preclinical testing stages, with experimental studies only starting to emerge from 2010. Stem cells can provide critical benefits to tissue engineered burns treatment by stimulating direct differentiation into skin tissue structures, and interacting with nearby cells to create a more inducive environment for regeneration. The therapeutic potential of stem cells arises from their ability to secrete regenerative cytokines, making them an attractive choice for treating chronic wounds [27]. In preclinical treatments of burn wound models, inclusion of stem cells has resulted in better wound healing by inducing improved granulation tissue formation, collagen deposition, healing speed, wound appearance, amount of scarring, presence of adnexal structures, regulation of inflammatory markers, and formation of vascular structures in the epidermal layer as well as its thickness and structure. By far the most popular type of stem cell being used in preclinical studies of burn wound repair are mesenchymal stromal cells (MSCs), commonly harvested from adipose tissue, bone marrow, and umbilical cord. MSCs play an important role in skin homeostasis and damage repair by promoting immune regulation, monitoring resident stem cells, and secreting growth factors to drive epithelialisation and neovascularisation [28, 29]. Other types of adult stem cells have proven to be useful for skin repair, such as those derived from hair follicle, dental pulp, and kidney. Pluripotent stem cells have also been used, but may raise several issues. Embryonic stem cells (ESCs) and foetal stem cells (FSCs) are associated with moral concerns and substantial legal restrictions, slowing down their applications in clinical wound healing [30, 31]. These and induced pluripotent stem cells (iPSCs) are also prone to teratoma formation due to their pluripotency depending on the efficiency of the differentiation protocol, raising potential safety concerns [32]. Hence, ESCs, FSCs and iPSCs have not been included in our analysis due to their current limitations in being used for clinical applications. In vitro studies have also been excluded, since studies involving animal models are more closely representative of the clinical performance of new burns treatment strategies.

The vast majority of studies using a stem cell-based approach for treating burn wounds are at the preclinical testing stage, and vary widely in their methods and outcomes. Over the past three years, only a few reviews have discussed tissue engineering methods for skin repair, with a primary focus on biomaterials [33], murine models [34], pluripotent stem cells [32], or immunomodulation [31] and other signalling pathways [30]. This review will cover the latest advances in tissue engineered solutions involving adult stem cells, tested in a variety of preclinical models. It will inform researchers and clinicians on the current progress in developing an ideal stem cell-based treatment for burn wounds, reflecting on a range of aspects including the type and source of stem cell, type of scaffold/matrix, animal model, graft type, type of wound injury, timeframe of treatment and analysis, and any proposed mechanisms.

### Methods

#### Literature search strategy

Selection of studies in this review was performed using the PRISMA scoping review protocol and checklist [35]. A comprehensive search of the electronic databases Embase, Medline, Scopus, and Web of Science was conducted for studies published since 1 January 2009, on using stem cell-based tissue engineering approaches to treat burn wounds in preclinical animal models. Specific search strategies used for each database are presented in the Supplementary Information.

#### Inclusion and exclusion criteria

The inclusion criteria for this review were peer-reviewed original research articles sourced from online databases, published between January 2009 and June 2021, in English language. The studies (1) must have tested a stem cell-based tissue engineering product, defined as a functional construct containing a specifically defined stem cell type and a supporting matrix or scaffold, (2) the purpose of the product must have been for skin regeneration or wound healing, (3) the product must have been used on a burn wound or the authors must have stated that it could be used to treat burns, and (4) the product must have been tested on an animal skin wound model.

The exclusion criteria were non-original research articles, articles published before 2009 or not in English language, and research where full text was not available including conference abstracts. Studies were also excluded if they (1) did not test a tissue engineered wound dressing or skin substitute (for example, placing stem cells directly onto a wound, or injecting stem cells into a wound), (2) did not incorporate a specifically defined type of adult stem cell (for example, precursor cells, progenitor cells, keratinocytes, stem cell-like cells, colony-forming units, ESCs, FSCs, iPSCs, growth factors, cytokines, or tissues that were said to contain stem cells without properly isolating and classifying them), (3) did not use the product for skin regeneration or state that it could be used for this purpose, (4) did not apply the product to burns or state that it could be used for this purpose, and (5) did not test the product in an animal skin wound model.

#### Study screening and reporting

The screening process followed a structure set out in the PRISMA flow chart [36]. The records of retrieved studies were imported into Endnote X8 for study screening. Title and abstract screening were performed, followed by full-text screening using the inclusion and exclusion criteria. Studies where the full text was not accessible were excluded. Data items extracted from articles included animal model, source of stem cell, type of stem cell, type of graft, skin wound model, timeframe of treatment, length of follow-up, type of scaffold/matrix, summary of findings, and proposed mechanisms.

### Results

**Fig. 1:**
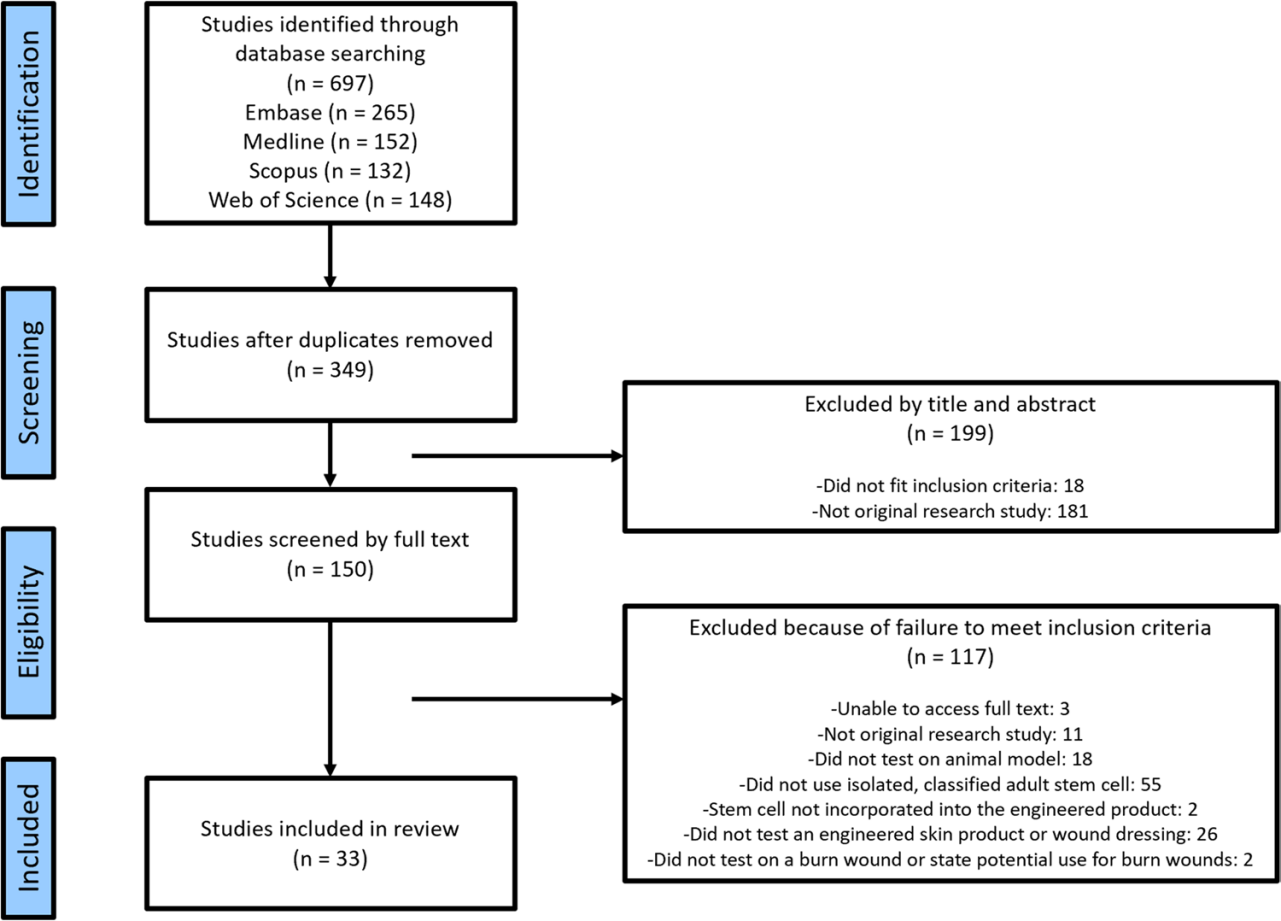
Flow chart for the study selection process

There were 697 potential studies identified from database searches. After removing duplicates, 349 studies were screened based on title and abstract. From these, 150 studies were included for full text screening, after removing articles that were not relevant to topic of this review, or were not original research articles. Of the 150 studies assessed by full text screening, 33 were eligible for inclusion in this review. The 117 that were excluded did not comply with the inclusion and exclusion criteria: 3 were unable to be obtained as full text articles, 11 were not original research studies, 18 were not tested on an animal model, 55 did not use a defined source of adult stem cells, 2 did not incorporate stem cells into the tissue engineered product, 26 did not test a tissue engineered product or wound dressing, and 2 did not test the product on a burn wound model or specifically state a potential use in burns. The study selection process is outlined in Fig. 1.

#### Main findings

A summary of the included studies is presented in Table 1, depicting the animal model, skin wound model, treatment timeframe, final endpoint, type of graft, type and source of stem cells used, type of scaffold/matrix, summary of findings, and proposed mechanisms. Where specified, a summary of results from the included studies on the time period for wound recovery (defined as when granulation tissue has begun to fill the wound), thickness of neo-skin formed in the treatment group(s), and incidence and nature of complications is presented in Table 2.

**Table 1:** Summary of the 33 studies included in this review

Reference	Animal model	Wound model + treatment timeframe	Final endpoint	Type of graft	Stem cells used	Scaffold/matrix + any other added substances	Summary of findings	Proposed mechanisms
Gholipour-Kanani, A., et al. (2012) [159]	Rat	2^nd^/3^rd^ degree thermal burn (immediate)	15 days	Xenogeneic	Human MSCs from umbilical cord	Electrospun chitosan-PVA nanofibrous scaffold	- Advanced granulation tissue formation and improved collagenous regeneration - Rapid and accelerated wound healing process	- Nanofibres attract fibroblasts to dermal layer, which excrete collagen and cytokines - Cells in scaffolds provide signals needed for tissue regeneration
Shokrgozar, M. A., et al. (2012) [160]	Rat	3^rd^ degree thermal burn (10d post-burn)	14 days	Allogeneic	Rat MSCs from upper intestinal adipose tissue	Crosslinked collagen-chitosan scaffold	- Wound healing process was effective and quicker - Faster regeneration of the dermal and epidermal layers after 14d	- Differentiation of MSCs to keratinocytes in vivo promotes wound healing
Natesan, S., et al. (2013) [161]	Rat	Full-thickness surgical wound (immediate)	16 days	Xenogeneic	Human debrided skin adipose stem cells	Collagen-PEG fibrin-based bilayer hydrogel	- Less wound contraction -Better dermal matrix deposition and epithelial margin progression - Formation of vascular network structures	- Fibrin gel promotes homeostasis, wound healing, tissue connection, angiogenesis and prevents infection - PEG-fibrin gel promotes MSC differentiation into vascular phenotypes in vitro
Zamora, D. O., et al. (2013) [162]	Rat	Full-thickness surgical wound (immediate)	16 days	Xenogeneic	Human debrided skin AdSCs	PEG-fibrin 3D gel	- Earlier collagen deposition and wound remodelling - Vessel-like structures appeared sooner - Increased amount of larger blood vessels	- Discarded skin AdSCs enhance angiogenesis by VEGF expression - Fibrin stimulates tissue and blood vessel growth
Gholipour-Kanani, A., et al. (2014) [163]	Rat	Two groups: 1. Full-thickness surgical wound 2. Full-thickness thermal burn (both immediate)	15 days	Xenogeneic	Human MSCs from umbilical cord	Electrospun poly(ε-caprolactone)-chitosan-PVA blend nanofibrous scaffold	- Treated wound areas were smaller -Scaffold showed better cell attachment, viability and compatibility	- MCSs secrete antimicrobial factors and stimulate phagocytosis by immune cells - MSCs express GF’s to promote granulation and epithelialisation - MSCs promote organised ECM deposition
Leiros, G. J., et al. (2014) [164]	Mouse	Full-thickness surgical wound (immediate)	70 days	Xenogeneic	Human HFSCs from occipital scalp, and immortalised human bulge stem cell-like Tel-E6E7	Porcine acellular dermal matrix + dermal papilla cells (DPCs) or dermal fibroblasts (DFs)	- HFSC/DPC induced a multilayered, stratified epidermis -HFSC/DPC favoured early neovascularisation and promoted neovascular network maturation - HFSC/DPC induced epidermal hair buds	- DPCs secrete VEGF and angiogenin - DFs or DPCs express cytokines or induce their expression by macrophages or other inflammatory cells recruited to the area
Rodrigues, C., et al. (2014) [73]	Rat	Dermal/epidermal surgical wound (immediate)	16 days	Allogeneic	Rat adipose-derived MSCs from dorsal region	Sodium carboxymethyl cellulose scaffold	- Increased proliferation of epithelial cells, epithelial thickness and cytokeratin expression	- MSCs regulate synthesis of collagen by releasing anti-fibrogenic molecules
Steffens, D., et al. (2014) [89]	Mouse	Full thickness surgical wound (immediate)	7 days	Isogeneic	Mouse MSCs from kidney	poly-D,L-lactic acid (PDLLA) nanofiber scaffold + spirulina biomass	- No microscopic difference between control and experimental groups -Macroscopic analysis showed better cicatrisation	- Spirulina aids differentiation of MSCs into cell types
Yang, Y., et al. (2014) [165]	Rat	Thermal burn (scald) (4d post-burn)	30 days	Allogeneic	Rat bone marrow MSCs from the femur and tibia	Fibrin glue	- Increased sebaceous glands - Hair follicle-like structures - Accelerated scald wound healing time	- Differentiation of MSCs into neotissues - MSCs exert paracrine effects on the wound
Guo, X., et al. (2016) [166]	Rat	Deep partial-thickness thermal burn (3d post-burn)	21 days	Allogeneic	Rat MSCs from tibia and femur bone marrow	Sterilised, decellularised porcine small intestinal submucosa (SIS)	- SIS and MSC seeded SIS accelerate burn wound closure: enhance granulation tissue formation, increase wound maturity and improve revascularisation - Increased blood vessel density - Accelerated proliferation of neo-epidermal cells	- SIS supports nutrient diffusion and enables adherence, growth and migration of seeded cells - MSCs induce angiogenesis from secretion of GFs and cytokines to alter behaviour of resident endothelial cells - MCSs increase proliferation of differentiated epidermal cells - MCS-derived exosomes may stimulate wound healing
Kong, Y., et al. (2016) [167]	Mouse	Full-thickness surgical wound (immediate)	9 days	Allogeneic	Mouse bone marrow stem cells	Chitosan/alginate nanomembrane	- Accelerated wound contraction and epidermalisation	- Chitosan/alginate membrane promotes cell adhesion, migration, proliferation and differentiation
Montanucci, P., et al. (2017) [168]	Mouse	Full-thickness surgical wound (immediate)	36 days	Xenogeneic	Human post-partum umbilical cord MSCs from umbilical cord Wharton Jelly	Human umbilical cord adult MSCs/fibrin-based scaffold	- Slower healing time for DE treated wounds - Improved wound appearance - Hair and subcutaneous gland budding	- MSC differentiation and paracrine signalling improves tissue repair - Human umbilical cord MSCs may stimulate murine myofibroblasts and induce their proliferation and differentiation
Motamed, S., et al. (2017) [169]	Rat	3^rd^ degree thermal burn (1d post-burn)	60 days	Xenogeneic	Human AdSCs from adipose tissue	Human amniotic membrane	- Accelerated healing rate - Significant reduction in wound surface measurements - Reduced inflammation	- Inflammation modulation and paracrine activation of host cells through GF secretion by AdSCs - AdSCs transdifferentiate towards endothelial and epithelial cells
Steffens, D., et al. (2017) [170]	Mouse	Full thickness surgical wound (immediate)	9 days	Xenogeneic	Human MSCs from dental pulp of extracted deciduous teeth	1. poly-D,L-lactic acid (PDLLA) 2. Laminin- functionalized poly-D,L-lactic acid scaffold (PDLLA/LAM) + keratinocytes	- Stimulated healing of skin - Reduced visual wound size with presence of laminin - In some animals, the epidermis formed throughout the length of the wound - Increased vascularisation - Reduced Inflammation	VEGF, SDF-1, TDF secretion
Alapure, B. V., et al. (2018) [123]	Mouse	Full thickness thermal burn (2d post-burn)	8 days	Allogeneic	Mouse MSCs from bone marrow	Arginine-based poly(ester amide) (Arg-PEA) and chitosan scaffold	- Accelerated wound closure - Promoted re-epithelialisation, granulation tissue formation and vascularisation - Reduced late phase inflammation	- Scaffold with and without MSCs induce high levels of IL-10, increase M2-like macrophage numbers, and reduce TNF-α - Scaffold can promote MSC production of angiogenic and/or regenerative GF, cytokines, and chemokines in wounds, including VEGF, IGF, HGF, IL-10
Burmeister, D. M., et al. (2018) [72]	Pig	Deep partial-thickness thermal burn (4d post-burn)	42 days	Allogeneic	Porcine AdSCs from the subcutaneous fat of the nape	PEG-fibrin-based hydrogels	- Increased size of blood vessels in graft and granulation tissue -Acceleration of angiogenesis - Dose-dependent effect on collagen deposition	- AdSCs release VEGF - AdSCs may promote vasodilation and increase blood flow
Edwards, N., et al. (2018) [171]	Mouse	Full-thickness surgical wound (immediate)	21 days	Xenogeneic	Human AdSCs from RoosterBio Inc	Electrochemically deposited collagen wound matrix (CWM)	- With or without AdSCs, the CWM showed excellent wound healing and regeneration -Increased granulation tissue formation and epidermal thickness	None
Gholipourmalekabadi, M., et al. (2018) [172]	Mouse	3^rd^ degree thermal burn (immediate)	28 days	Allogeneic	Mouse adipose tissue- derived MSCs from inguinal fat pad	Decellularised human amniotic membrane ± electrospun nanofibrous silk fibroin	- Accelerated wound healing and neovascularisation - Reduced scarring	- MSCs and silk fibroin accelerate wound healing through early re-epithelialisation and ECM formation - MSCs modulate scarring by decreasing the inflammatory response, collagen deposition and inducing MMP expression - Increased MMPs promote neovascularisation
Forbes, D., et al. (2019) [173]	Mouse	Full-thickness surgical wound (immediate)	14 days	Allogeneic	Mouse AdSCs from subcutaneous fat	Liquid dermal scaffold (LDS): Collagen-GAG scaffold crosslinked containing PVA hydrogel	- LDS with and without AdSCs accelerates healing - LDS with and without AdSCs result in increased angiogenesis - LDS with AdSCs result in thicker epidermis with higher collagen content	- AdSCs increase VEGF-α and HGF - AdSCs stimulate fibroblasts to increase pro-collagen gene expression via exosomes or through de novo differentiation of AdSCs into fibroblasts
Kakabadze, Z., et al. (2019) [101]	Rat	3^rd^ degree radiation burn (20d post-burn)	90 days	Allogeneic	Rat bone marrow MSCs from femur	Decellularised and lyophilised human amniotic membrane grafts	- Healing rate increased	- MSCs secrete VEGF, GCSF, HGF, monocyte chemotactic protein-1, IL-6, and TGF β1 - Human amniotic membrane contains EGF, BFGF, KGF, VEGF, TGF-α, TGF-β, PDGF, HGF and NGF
Koo, M. A., et al. (2019) [174]	Mouse	Full-thickness surgical wound (immediate)	21 days	Xenogeneic	Human bone marrow MSCs from bone marrow	Hematoporphyrin-incorporated polyketone film, to make single or multiple layer cell sheets	- Promoted angiogenesis and skin regeneration at site of wound - Neovascularisation in the subcutaneous layer	- MSCs increase VEGF - MSCs may differentiate into endothelial cells
Nazempour, M., et al. (2019) [175]	Rat	3^rd^ degree thermal burn (immediate)	21 days	Xenogeneic	Human WJSCs from umbilical cord tissue	Human skin acellular dermal matrix	- Improved angiogenesis and granulation tissue formation - Decreased inflammation, necrosis and fibrosis -Decreased wound size	- MSCs downregulate inflammatory markers, upregulate anti-inflammatory markers, and increase local anti-inflammatory cytokines - MSCs secrete extracellular vesicles which downregulate IL-6 and nitric oxide synthase, and increase IL-10 and ATP
Samberg, M., et al. (2019) [176]	Rat	Full-thickness surgical wound (immediate)	12 days	Xenogeneic	Human AdSCs from discarded abdominoplasty skin tissue	Modified PEG platelet-rich plasma (PRP) hydrogel	- Decreased granulation tissue formation - Faster wound closure - Increased angiogenesis	- AdSCs express vascular specific genes of α-SMA, VEGF, Angpt-1, and Angpt-2 - AdSCs upregulate VEGF, PLGF and sFlt-1
Zhang, Y. Z., et al. (2019) [100]	Mouse	Radiation burn (immediate)	21 days	Allogeneic	Mouse adipose-derived MSCs from bilateral groin tissue	Atelocollagen matrix	- Smaller wound sizes - Accelerated wound healing - Accelerated angiogenesis - Increased collagen production - Decreased inflammation	- AdSCs upregulate VEGF - AdSCs downregulate IL-1β
Hashemi, S. S., et al. (2020) [177]	Rat	3^rd^ degree thermal burn (1d post-burn)	14 days	Xenogeneic	Human WJMSCs from amniotic and umbilical cord	Decellularised human amniotic membrane	- Improved healing rate -Early re-epithelialisation - Absence of inflammation	- WJMSCs promote paracrine signalling
Liu, F., et al. (2020) [50]	Mouse	Full-thickness surgical wound (immediate)	21 days	Xenogeneic	Rat HFSCs from skin	Human acellular amniotic membrane (hAAM)	- Promoted wound healing -Hair follicle formation and angiogenesis of tissue around hair follicle	- rHFSCs-hAAM composite promotes neovascularisation - rHFSCs assist the formation of follicle-like tissues and the vascularisation of adjacent follicle-like tissues
Cheng, R. Y., et al. (2020) [178]	Pig	Full-thickness thermal burn (2-3d post-burn)	28 days	Xenogeneic	Human MSCs from umbilical cord Wharton’s Jelly	Fibrinogen and hyaluronic acid solution combined with cross-linker solution containing thrombin and hyaluronic acid	- Superior healing - Reduced inflammation, scarring and contraction - Superior restoration of overall epidermal thickness and dermal collagen density	- Elevated CD31 + expressing endothelial cells of vessels - Lower levels of expression of M2 macrophage marker CD163 and pan-inflammatory marker CD11b - Reduced number of SMA expressing myofibroblasts
Lu, T.-Y., et al. (2020) [71]	Rat	Partial-thickness thermal burn (immediate)	14 days	Xenogeneic	Human AdSCs from subcutaneous fat	Gelatin/microbial transglutaminase hydrogel	- Accelerate wound healing - Increased epidermal thickening - Increased collagen synthesis and deposition - Promotion of neovascularisation	- AdSCs release GFs such as PDGF, VEGF, and bFGF which promote angiogenesis in wound healing - AdSCs can promote endothelial cells proliferation leading to microvessel formation - Cell spheroids induce collagen synthesis
Paramasivam, T., et al. (2020) [138]	Rat	Thermal burn (3 days post-burn)	28 days	Allogeneic	Rat bone marrow MSCs from femur and tibia	Porcine acellular urinary bladder scaffold + PDGF-B gene added to MSCs	- Early extracellular matrix deposition - Promoted healing with neovascularisation and neo tissue formation - reduced scar formation	- PDGF-B induces migration of repair cells to wound, and stimulates proliferation of repair cells - Increased production of PDGF-B gene simultaneously increases production of VEGF
Thanusha, A. V., et al. (2020) [179]	Rat	2^nd^ degree thermal burn (1 day post-burn)	28 days	Xenogeneic	Human MSCs from bone marrow	Gelatin GAG foam matrix	- Scaffold ± MSCs increased rate of wound contraction - Increased epithelisation and collagen formation	- MMP-2 presence promotes signalling - Seeded MSCs might be unable to express their activity in the burnt portion due to the lack of nutrients to grow and leads to a mortal state
Eylert, G., et al. (2021) [74]	Pig	Full-thickness thermal burn (2d post-burn)	28 days	Xenogeneic	Human MSCs from umbilical cord Wharton’s Jelly	Collagen-based dermal regeneration template Integra®	- Less scarring - Faster epithelialisation - Reduced inflammation - Increased collagen formation - Increased neovascularisation - Reduced fibrosis	- MSCs reduce hypoxia-induced apoptosis - MSCs upregulate inflammation
Barrear, J. A., et al. (2021) [180]	Mouse	Partial thickness thermal burn (5d post burn)	29 days	Allogeneic	AdSCs from inguinal fat pads of mice	Collagen-pullulan hydrogel	- Accelerated re-epithelisation - Increased vascularity - Reduced scarring	- AdSCs increase expression of MCP-1, VEGF and SDF-1, which increases neovascular response - AdSCs decreased TIMP1 and TNF-α reducing inflammation
Roshangar, L. et al. (2021) [181]	Rat	Full thickness thermal burn (1d post-burn)	21 days	Allogeneic	AdSCs from inguinal and pararenal region of rat	3D printed collagen and alginate bioink	- Increased wound closure - Decreased inflammation - Increased epithelisation - Reduced scar formation	- Differentiation of AdSCs to keratinocytes assists the reformation of epithelium across the implanted scaffold

AdSCs: adipose-derived stem cells, bFGF: basic fibroblast growth factor, ECM: extracellular matrix, EGF: epidermal growth factor, GAG: glycosaminoglycan, GCSF: granulocyte colony-stimulating factor, GF: growth factor, HFSCs: hair follicle stem cells, HGF: hepatocyte growth factor, IL: interleukin, KGF: keratinocyte growth factor, MCP: monocyte chemoattractant protein, MMP: matrix metalloproteinase, MSCs: mesenchymal stem cells, NGF: nerve growth factor, PDGF: platelet-derived growth factor, PEG: poly(ethylene glycol), PVA: poly(vinyl alcohol), SDF: stromal cell-derived factor, SMA: smooth muscle actin, TGF: transforming growth factor, TIMP: tissue inhibitor of metalloproteinase, TNF-α: tumour necrosis factor alpha, VEGF: vascular endothelial growth factor, WJSCs: Wharton’s jelly stem cells.

**Table 2:** Summary of results from the included studies on time period for wound recovery (defined as when granulation tissue has begun to fill the wound), thickness of neo-skin, and incidence and nature of complications (unspecified data indicated by -)

Reference	Animal model	Time period for start of wound recovery (days)	Thickness of neo-skin in treatment group(s)	Complications	
				Number of animals	Nature of complications & group(s) affected
Gholipour-Kanani, A., et al. (2012) [159]	Rat	10	-	-	-
Shokrgozar, M. A., et al. (2012) [160]	Rat	14	-	-	-
Natesan, S., et al. (2013) [161]	Rat	8	-	-	-
Zamora, D. O., et al. (2013) [162]	Rat	8	-	2	Inflammation, infection, redness (but no mortality): Saline control group only
Gholipour-Kanani, A., et al. (2014) [163]	Rat	10	-	-	-
Leiros, G. J., et al. (2014) [164]	Mouse	14	-	-	-
Rodrigues, C., et al. (2014) [73]	Rat	8	-	-	-
Steffens, D., et al. (2014) [89]	Mouse	-	-	15	Ulceration, inflammation, fibrosis (but no mortality): All control and treatment groups
Yang, Y., et al. (2014) [165]	Rat	14	-	-	-
Guo, X., et al. (2016) [166]	Rat	7	-	-	-
Kong, Y., et al. (2016) [167]	Mouse	7	Granulation tissue mean thickness: 600 μm		-
Montanucci, P., et al. (2017) [168]	Mouse	15	-	-	-
Motamed, S., et al. (2017) [169]	Rat	7	-	32	Dermis showed oedema, mild to moderate acute and chronic inflammatory cell infiltration and fibrosis, and partial destruction of dermal appendages: All control and treatment groups
Steffens, D., et al. (2017) [170]	Mouse	-	-	-	-
Alapure, B. V., et al. (2018) [123]	Mouse	8	-	-	-
Burmeister, D. M., et al. (2018) [72]	Pig	10	-	-	-
Edwards, N., et al. (2018) [171]	Mouse	7	Epidermis mean thickness: 60 μm	-	-
Gholipourmalekabadi, M., et al. (2018) [172]	Mouse	14	-	-	-
Forbes, D., et al. (2019) [173]	Mouse	14	-	-	-
Kakabadze, Z., et al. (2019) [101]	Rat	14	-	15	Oedema and neutrophilic tissue infiltration: Untreated injury group only
Koo, M. A., et al. (2019) [174]	Mouse	14	Epidermis/dermis mean thickness: 80/590 μm (cell suspension), 50/280 μm (1 layer cell sheet), 30/230 μm (3 layer cell sheet)	-	-
Nazempour, M., et al. (2019) [175]	Rat	21	-	-	-
Samberg, M., et al. (2019) [176]	Rat	8	-	-	-
Zhang, Y. Z., et al. (2019) [100]	Mouse	7	-	-	-
Hashemi, S. S., et al. (2020) [177]	Rat	7	-	32	Mild to moderate haemorrhaging: All control and treatment groups Mild inflammation: Scaffold alone group
Liu, F., et al. (2020) [50]	Mouse	7	-	-	-
Cheng, R. Y., et al. (2020) [178]	Pig	-	-	-	-
Lu, T.-Y., et al. (2020) [71]	Rat	14	-	-	-
Paramasivam, T., et al. (2020) [138]	Rat	7	-	-	-
Thanusha, A. V., et al. (2020) [179]	Rat	14	-	-	-
Eylert, G., et al. (2021) [74]	Pig	28	Epidermis median thickness: 189 μm (40,000 cells/cm^2^), 157 μm (200,000 cells/cm^2^), 131 μm (400,000 cells/cm^2^)	-	-
Barrear, J. A., et al. (2021) [180]	Mouse	10	-	-	-
Roshangar, L. et al. (2021) [181]	Rat	21	-	-	-

**Fig. 2:**
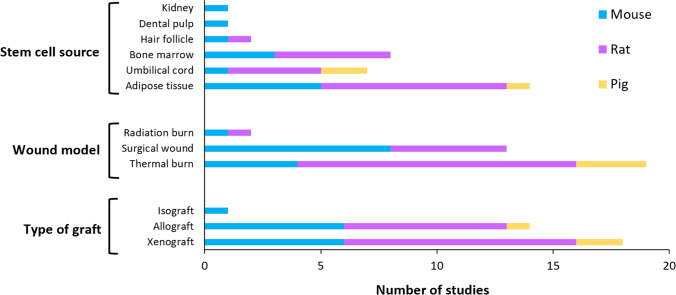
Sources of stem cells, wound model, and type of graft used in the included studies, further categorised by the frequency by which they were applied in different animal species (mouse, rat and pig)

The sources of stem cells, wound model, and type of graft used in the included studies are shown in Fig. 2. Among the 33 studies, 17 used rats, 13 used mice, and 3 used a porcine model. Studies using a mouse model included the widest variety of stem cell sources. MSCs were the most commonly used stem cell type among the included studies, which were often derived from adipose tissue, umbilical cord, or bone marrow. The most common type of graft applied across all animal models was a xenograft, consisting of a product containing stem cells derived from an animal of a different species from the graft recipient. The most common wound model used across all animal models was thermal burn, which was the most popular choice in rat and porcine studies, although surgical wound was more popular among mouse studies.

**Fig. 3:**
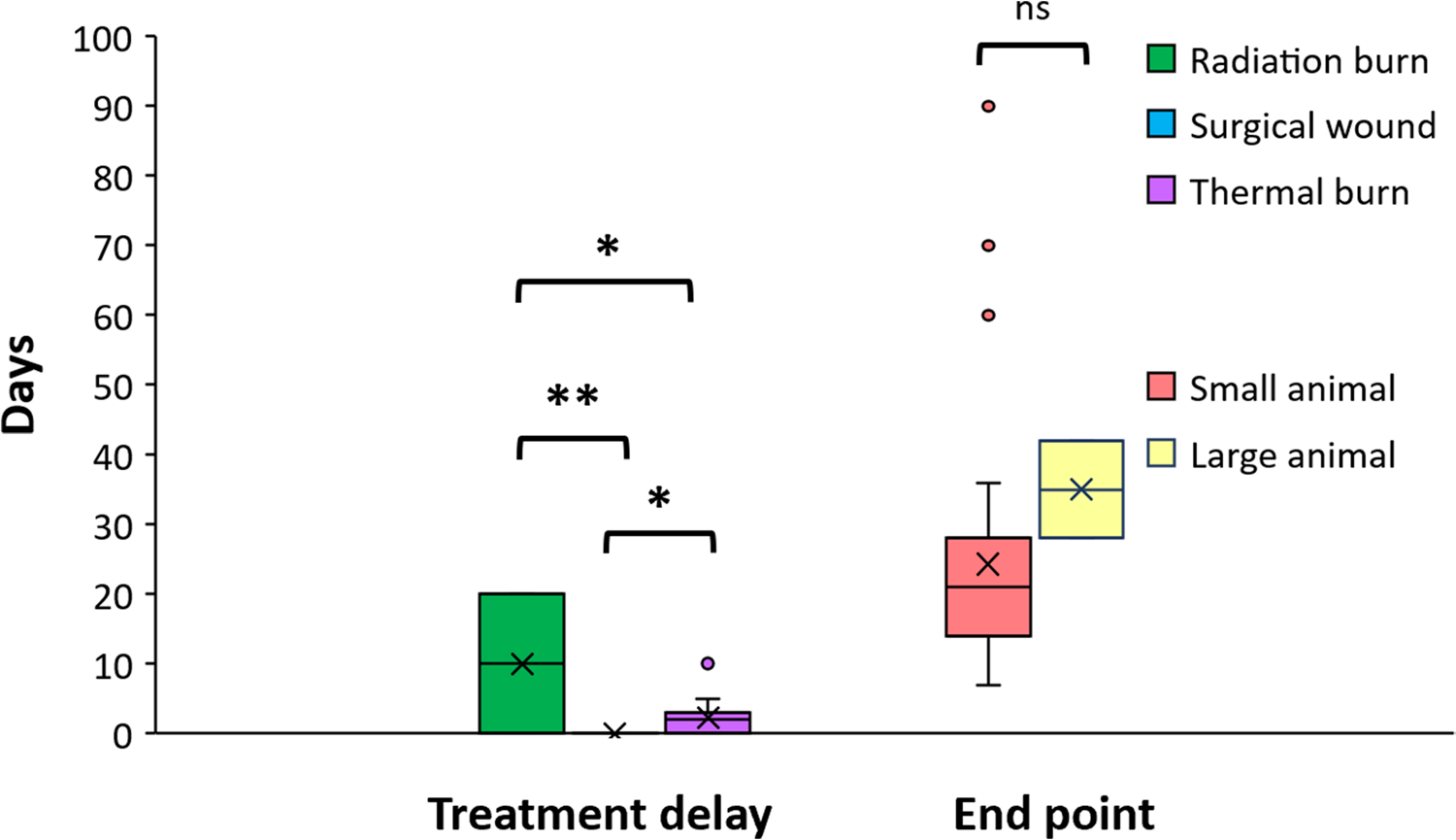
The number of days in treatment delay after wound model creation, and in assessing the end point after applying treatment for the included studies. *p < 0.05, **p < 0.005, ns = not significant

The number of days in treatment delay after wound model creation, and in assessing the end point after applying stem cell-based treatment for the included studies are shown in Fig. 3. All studies using a surgical wound model applied treatment immediately after inflicting the injury, while studies using thermal and radiation wounds varied in the number of days between wound creation and treatment, with the longest delay being 20 days for a radiation burn wound. The included studies varied in the end time point of analysis, with many having multiple time at which data was collected. In small animals (rats and mice), the final endpoints ranged between 7 to 90 days after treatment, while in large animals (pigs) longer follow-up periods were generally used, with endpoints ranging from 28 to 42 days.

A wide variety of scaffolds and matrices were used to aid stem cell treatment in the included preclinical studies, as shown in Table 3. The biomaterials used included a wide range of natural, synthetic, and commercial materials. A number of studies also included additional substances such as other cell types or bioactive factors along with the scaffolds.

**Table 3:** Types of scaffold materials and other substances used in the included studies delivered together with stem cells

Natural	Synthetic	Commercial	Additional substances
Acellular amniotic membrane [50, 101, 169, 172, 177] Acellular dermal matrix [164, 175] Alginate [167, 181] Atelocollagen [100] Chitosan [123, 159, 160, 163, 167, 181] Collagen [160, 161, 171, 173] [180] Decellularised bladder [138] Decellularised small intestine submucosa [166] Fibrin [72, 161, 162, 165, 168] Fibrinogen [178] Gelatin [71] Glycosaminoglycan [173, 179] Hyaluronic acid [178] Platelet-rich plasma [176] Silk fibroin [172] Sodium carboxymethyl cellulose [73] Thrombin [178]	Arginine-based polyester amide [123] Polycaprolactone [163] Poly-D,L-lactic acid [89, 170] Polyethylene glycol [72, 161, 162, 176] Polyketone [174] Polyvinyl alcohol [159, 163, 173]	Integra® [74]	Dermal fibroblasts [164] Dermal papilla cells [164] Hematoporphyrin [174] Keratinocytes [170] PDGF-B gene [138] Spirulina biomass [89]

## Discussion

Burn wounds can have severe impacts on quality of life, and current clinical treatments still face many challenges in restoring skin that is anatomically and functionally similar to native tissue, particularly for complex scenarios such as full-thickness or large area burns. Tissue engineering strategies incorporating stem cells have recently opened new doors for the effective treatment of burn wounds. Studies in preclinical models over the last 10 years, captured in this review, demonstrate substantial progress and highlight the prospect of stem cell-based tissue engineered skin constructs becoming a reality in the treatment of clinical burn injuries. In this section, we offer critical insights into different aspects of information reflected in the included studies, providing the latest updates in this exciting field of research.

### Animal models

In our analysis, rats were the most commonly used animal model for burn wound treatment, followed very closely by mice. The primary advantages for using rats in burns studies are their availability and cost-effectiveness [37]. Rats also share several physiological similarities with humans, the most relevant being that rat skin is composed of the dermis and epidermis [38]. However, the primary wound healing method in rats is wound contraction, instead of re-epithelisation in humans [37]. The reduced healing time in rats allows researchers to study wound healing mechanisms more rapidly and efficiently. However, rapid healing times and even spontaneous healing can also prevent the risk of sepsis or immunosuppression that are generally seen in larger animal models, reducing the relevance of rats as a model for predicting the outcome of clinical burn wound treatment. Likewise, although mouse models mimic many aspects of human responses to burn injury, there are substantial differences that need to be considered [39]. Dermal and epidermal thickness, scar formation and glucose metabolism post-burn injury differ greatly between mice and humans. Thus, although small animal models confer economic advantages, have rapid reproduction rates, and reduce the time required for study, they have limited translational relevance to humans.

Three of the included studies used pigs as a burn wound model, which have great anatomical and physiological similarity to humans, including in skin structure and response to therapeutics, dermal physiology, transdermal toxicology, wound healing, and neurophysiology [40]. Both humans and pigs have a thick epidermis, 50–120 μm in humans and 30–140 μm in pigs [40, 41]. In contrast to small and loose-skinned animals such as rodents, pigs and humans do not possess a panniculus carnosus, meaning that both species have a similar distribution of skin blood vessels. Both species also have sparse body hair, which is important since hair follicles impact the process of re-epithelisation. Due to the larger size of pigs, multiple treatments can be compared within the same animal, which reduces inter-individual variability [42]. However, this is not applicable for treatments which induce systemic effects. While porcine models are an optimal choice from a physiological perspective, they have higher costs and special housing requirements, as well as increased ethical concerns over their use [43].

### Source of stem cells

Stem cells used in the included studies were harvested from a variety of sources including adipose tissue, bone marrow, umbilical cord, hair follicle, and dental pulp. These sources differed with respect to their ease of access and feasibility of use in treating burn wounds.

Conventional bone marrow-derived MSCs (BM-MSCs) have limited availability compared to some other sources such as adipose tissue, but treatments utilising BM-MSCs show potential to enhance skin regeneration, for example, by allowing the formation of a thicker epidermal layer and increased cell proliferation, collagen synthesis, and angiogenesis [44]. When obtaining BM-MSCs, aspiration volume directly correlates with cellular yield. Lower aspiration volumes of 10 and 20 mL have been shown to contain a lower concentration of nucleated cells and yield a lower number of MSCs [45]. In comparison, adipose tissue has been considered a more attractive source for harvesting stem cells, since subcutaneous adipose tissue can be easily accessed and repeatedly sampled, and the enzyme-based isolation procedures are not complicated [46]. The greatest advantage is the high quantity of cells that can be isolated, for instance, it is possible to obtain up to 3.5 million AdSCs from 1 g of adipose tissue [47]. However, although using liposuction as a standard procedure to collect adipose tissue is relatively safe and has minimal discomfort for the patient, this harvesting method may negatively affect the amount and viability of isolated AdSCs. The umbilical cord presents a reliable, accessible, and non-controversial source of stem cells. Applying umbilical cord-derived stem cells in burn wound models has been found to thicken the epidermis, increase the amount of dermal ridges, and create a better alignment of collagen fibres, demonstrating their active participation in skin wound healing through regenerative processes [48]. A novel method of isolating these stem cells involves cryopreserving cells from fresh umbilical cord post-delivery, in autologous cord plasma, to effectively reduce the risk of prion or virus contamination [49]. However, unlike for bone marrow and adipose tissue, ready access to umbilical cord as a stem cell source is more limited. While these are the predominant sources of stem cells considered for treating burn wounds, less utilised sources such as hair follicles and dental pulp also have useful attributes.

Hair follicle stem cells (HSFCs) are involved in the formation of new hair follicles, epithelisation of wounds, and promotion of vascularisation in newly formed skin [50]. Although their original purpose is hair regeneration in response to skin injury, HFSCs are recruited at the site of a burn injury to differentiate into cells that assist in repairing the damaged epithelium [51]. Regulatory T-cells (Tregs) modulating localised inflammation help to promote HFSC differentiation, thereby contributing to skin-barrier regeneration. HFSCs also have multiple advantages including their abundant source, easy sampling and low tissue damage during the sampling procedure, high proliferative capacity and differentiation potential, and the blatant lack of ethical issues [50]. On the other hand, dental pulp stem cells (DPSCs) have potential as a stem cell source as they have been shown to generate mineralised tissue and extracellular matrix in xenograft models [52]. Current isolation methods for DPSCs include the explant method and the enzymatic digestion of pulp tissue method, but these techniques still need to be improved to achieve optimal proliferative capacity of cells, karyotypic stability, and clinal translatability. DPSCs can be implanted in chitosan, collagen or composite biomaterial scaffolds to induce tissue regeneration, and have shown positive results in regenerating periodontal tissue and skin lesions caused by burns. Their regenerative capacity in a burn wound repair model has been suggested to match stem cells derived from bone marrow and adipose tissue [53].

### Type of stem cells used and mechanisms of action

MSCs were the most common type of stem cells used in the included studies. They are a heterogeneous population that commonly refer to adult stem cells capable of differentiating into connective tissues including bone, muscle, cartilage, and fat [54]. MSCs are a popular choice for use in skin tissue regeneration due to a range of beneficial properties, and were the most commonly chosen stem cell type among the included studies in this review. They can be harvested from a wide variety of tissues such as bone marrow, adipose tissue, umbilical cord, and even menstrual blood. The main beneficial functions of MSCs in the context of skin repair include anti-inflammatory and immune-modulation effects [55–57], angiogenesis [58], and promotion of cell proliferation [59].

MSCs are known to migrate to wound sites, differentiate, and regenerate lost tissue by regulating cellular responses to injury through paracrine signalling [60]. Experimental studies have shown that MSCs can coordinate inflammatory responses following tissue injury. For instance, MSCs secrete multiple inflammatory modulators including nitric oxide (NO), indoleamine 2,3-dioxygenase (IDO), prostaglandin E2 (PGE2), interleukin(IL)-10 and TNF-alpha-stimulated gene/protein 6 (TSG-6) [61]. MSCs can also facilitate wound healing by increasing the secretion of anti-inflammatory cytokines such as IL-10, which results in reduced levels of inflammatory IL-6 and IL-8, as well as decreased collagen production and hence reduced fibrosis [62]. Other beneficial interactions of MSCs include promoting the production of anti-inflammatory IL-35, PGE2 which reduces natural killer cell proliferation and T-cell migration, vascular endothelial growth factor (VEGF) which promotes angiogenesis, and hepatocyte growth factor (HGF) which is involved in downregulating fibrosis and increasing cell recruitment in the wound bed [62]. MSCs can also influence wound healing by decreasing the levels of pro-inflammatory cytokines including tumour necrosis factor (TNF)-α and interferon (IFN)-γ [63]. In the remodelling phase of wound healing, MSCs produce transforming growth factor (TGF)-β3 and keratinocyte growth factor (KGF), as well as regulate the activity of matrix metalloproteinases (MMPs) and tissue inhibitors of metalloproteinases (TIMPs), and collagen deposition [60, 64, 65].

The anti-inflammatory and immune-modulatory properties of MSCs make allogeneic transplantation possible [66]. Allogeneic human MSCs and some xenogeneic MSCs can avoid acute immune rejection through the expression of factor H and other complementary proteins [67]. They possess the ability to block neutrophil function by supressing the oxidative bursts of both resting and activated neutrophils, while simultaneously preserving neutrophil phagocytic chemotactic functions. Cytokines expressed by MSCs have been shown to play a critical role in immune-modulation mechanisms [68]. Quiescent MSCs mainly produce the immune-regulatory cytokines TGF-β and IL-10, whereby TGF-β inhibits IL‐2, major histocompatibility complex II (MHC‐II), and co‐stimulatory factor expression in dendritic cells (DCs) and T-cells, while IL-10 inhibits antigen-presenting cell maturation and suppresses T helper 17 cell (Th17) generation [68].

MSCs also produce a wealth of bioactive trophic factors, which simulate adjacent parenchymal cells to kick-start the process of repairing damaged tissue [69]. In wound repair, MSCs play an important role in promoting angiogenesis, a complex process controlled by both pro-angiogenic and anti-angiogenic factors. The ability of MSCs to mediate angiogenesis has been shown in a study where BM-MSCs engrafted in a skin wound released pro-angiogenic factors [70]. Human AdSCs have also been shown to secrete growth factors such as platelet-derived growth factor (PDGF), VEGF, and TGF-β, which are directly involved in angiogenesis and wound healing, and resulted in increased microvessel formation in a murine burn model [71]. A study using the porcine model also confirmed that AdSC delivery can accelerate angiogenesis in a dose dependent manner in deep partial thickness burns [72].

MSCs play a primary role in promoting cell proliferation and enhancing overall tissue repair. Cell cycle analysis and anti-proliferating cell nuclear antigen (PCNA) staining in a rat skin expansion model showed that AdSC-treated injuries had significantly higher percentages of cell proliferation compared to fibroblast-treated injuries [18]. The proliferating cells were predominantly observed at the stratum basale and hair follicles. In another study using a rat skin wound model, regions treated with AdSCs showed increased rates of cell proliferation in granulation tissue. This was thought to be due to the influence of AdSCs on activated macrophages, which released significant amounts of fibroblast growth factor (FGF), a cytokine directly involved in the epithelial proliferation process [73]. The majority of studies included in this review observed that MSCs combined with a biomaterial scaffold have the capacity to accelerate wound healing, promote re-epithelisation, and induce skin tissue regeneration.

### Dose of stem cells

Although an interesting point of investigation, only one of the include studies compared the effectiveness of using different doses of MSCs in wound healing, by incorporating them into a dermal regeneration template [74]. This porcine study used umbilical cord MSCs, and surprisingly found that a low dose range of 200–40,000 cells/cm^2^ stem cells was the most effective in regenerating full-thickness burn excised wounds. This was followed by a middle dose range of 2–4 × 10^5^ cells/cm^2^, and lastly a high dose of 2 × 10^6^ cells/cm^2^. The lower doses proved more effective in several aspects of skin repair, including accelerated wound healing, reduced scarring, and enhanced neovascularisation. Moreover, epidermal thickness was observed to be highest in the low dose group and decreased with increasing cell dose. These findings are potentially paradigm shifting among current beliefs in skin tissue regeneration, as previous studies hypothesised that a larger dose of stem cells was required to enhance skin healing [75–81]. To explain these findings, it was hypothesised that excessive amounts of transplanted stem cells might proliferate to the maximum capacity and consume all available resources, leading to possible nutrient deficiency and hypoxia in the wound environment that then catalysed cell death [74].

### Cell culture method: 2D vs 3D

MSCs cultured in 3D have the characteristics of enhanced differentiation capacity, upregulated pluripotency marker gene expression, and delayed replicative senescence [82]. Although there is no research to directly compare the effects of 2D versus 3D culture on burn wound healing, studies on other types of tissue regeneration show promising results. An in vitro study investigating the therapeutic effects of 3D spheroids formed from human MSCs for acute kidney injury showed that 3D culture enhanced the production of extracellular matrix (ECM) proteins including collagen I, fibronectin and laminin, when compared to cells cultured in 2D [83]. The 3D spheroids also exhibited stronger anti-oxidative and anti-apoptotic properties. When injected into the kidney of rat models, 3D cultured cells were more effective in protecting against apoptosis, reducing tissue damage, enhancing vascularisation, and improving overall renal function than 2D cultured cells. Another study examined the effects of human MSC secretome on corneal wound healing in rabbits [84]. Concentrations of bioactive factors such as HGF and ICAM-1 increased by up to five fold in the secretome produced by MSCs cultured on 3D fibre matrices compared to those on 2D culture dishes. The 3D cultured MSCs were effective at facilitating wound healing in corneal fibroblast cells and explanted corneas.

3D bioprinting is a recent technological advance that can be used to produce 3D cellular or tissue structures, possessing the advantages of high resolution, flexible operation, repeatable printing, and high-throughput output, making it an appealing option for generating bioactive constructs for the clinical treatment of skin burns [85]. Since the first 3D bioprinting technology was reported, tissue engineering has made great progress in this realm, with the field now progressing towards printing mini-sized organs and tissues [86]. Using 3D bioprinting, different types of cells can be deposited at specific locations to form multilayer structures and build anatomically-similar tissues [87]. In an example where 3D bioprinting was applied in skin tissue engineering, a dermal-derived ECM (dECM) bioink was used to tackle the rapid degradation and high shrinkage seen in traditional collagen-based bioinks. The printed mixture of adipose tissue-derived MSCs and endothelial progenitor cells, together with skin-derived dECM was used to produce pre-vascularised skin grafts, which effectively accelerated skin healing in a mouse excisional wound model [88].

### Type of graft

All included studies except one used allogeneic or xenogeneic grafts. One study used isogeneic cells from genetically identical clones [89].

Autologous skin grafts have remained the standard of care for clinical skin reconstruction and wound coverage, typically involving the use of split-thickness skin grafts (STSGs) and full-thickness skin grafts (FTSGs). Although both have been known to provide good healing outcomes in patients, they are limited by certain drawbacks. STSGs have lower levels of elastin compared to FTSGs and are unable to regenerate full-thickness skin, often undergoing significant contraction following placement [90]. While STSGs can cover large wound areas on the recipient, a variety of donor site morbidities can occur including scarring, chronic pain, and infection [91]. FTSGs are more preferable for skin repair since they contain all skin layers as well as the regenerative appendages found in each cutaneous compartment [90]. However, FTSGs have a higher metabolic demand and are limited by the lack of donor skin, restricting their application to smaller wounds. Interestingly, a recently developed autologous homologous skin construct (AHSC) technology makes use of the patient’s innate skin regeneration potential to generate full-thickness skin, including all dermal and epidermal components [92]. In this case, a 10-year-old boy who suffered from a large upper torso burn wound was treated with STSGs and developed painful, functionally limiting scar contractions. As alternative treatment, a 17.5 cm^2^ section of skin was harvested from the groin and sent to be manufactured into AHSC at a biomedical facility. The skin construct was applied to a 200 cm^2^ wound immediately following excision. The AHSC displayed 100% graft take, as well as initial postoperative epithelialisation and re-pigmentation which progressed to complete epithelial coverage at 8 weeks. At 11 months, the regenerated skin restored range of movement and showed no adverse scarring, suggesting the potential of this technology in paediatric burn reconstruction.

Allogeneic skin grafts catalyse potent immune responses involving both the innate and adaptive immune system, and their clinical applications need to rely heavily on effective immunosuppression in the recipient [93]. The immunocompromised state of patients with severe burn injuries receiving allogeneic skin grafts may result in delayed rejection, secondary infections, and increased scarring. In sandwich grafting for burn treatment, the skin allograft functions as a biological dressing that sits on top of a widely meshed autograft, to protect the wound bed in the interstices of the autograft [94]. Naturally, the allograft would separate from the wound bed due to gradual rejection, allowing the underlying autograft to complete the process of epithelisation. However, another major concern of using skin allografts is the risk of disease transmission. While the reported rates of disease transmission are sporadic and low, microbial testing is crucial to ensure the safety of the allografts.

Xenogeneic materials such as porcine or fish skin can be used for temporary skin coverage, particularly in large scale or severe burns to stabilise the patient until they can be treated by autologous skin grafting [93]. Xenogeneic grafts contain antigens which are recognised by the immune system as foreign, which can lead to biochemical failure of the graft or even host organism death [95]. For clinical application, chemical treatments may be used that crosslink proteins within xenogeneic tissue, but this may not completely mask important antigens [93]. Although a recent Phase II clinical trial has suggested that a xenograft dressing could achieve re-epithelialization in burn wounds even without autologous split-thickness skin grafting [96], xenografts currently can only be considered a temporary solution for the clinical care of patients with severe burns.

### Burn wound model

The types of preclinical burn models used in the included studies can be divided into thermal burn, radiation burn and surgical wound. Thermal burn was the most common injury used in animal models to test the effectiveness of tissue engineered constructs in burn wound healing. This is because thermal burns from fire, flames or scalds account for approximately 80% of all reported burns [97]. The metabolic response in burn patients following thermal injury is biphasic, with an initial ebb phase and then a hypermetabolic and catabolic flow phase [98]. The increased metabolic rate results from evaporative heat loss from the burn wound, as well as a central effect of inflammation on the hypothalamus. A study on the standardisation of thermal injuries in the rat model validated the same hypermetabolic response induced by thermal injury that is generally associated with severe burns [99]. Skin samples confirmed that animals receiving thermal burn wounds sustained skin injury across all layers, including complete epidermal destruction and thermal coagulative damage. Burned hair follicles also displayed distinct cellular damage and cytoplasmic swelling.

Surgical wounds were the second most common injury type among the included studies. Surgical wounds are typically created under sterile conditions, and do not result in the same immediate pathophysiological effects seen in conventional burns. Therefore, although surgical skin wound models are more consistent and controlled, they are less representative of clinical burn pathology and responses to treatment due to inherent differences in physiological responses.

Radiation burns are clinically far less common than thermal, electrical and chemical burns, with very different pathophysiological responses. Only 2 of the included studies used radiation burn in their animal models [100, 101]. Compared to thermal skin burns, radiation burns are characterised by necrosis, paroxysmal and chronic pain resistant to opiates, as well as uncontrolled, successive inflammatory waves [102]. Ulceration and necrosis may extend to deep dermal and underlying muscle structures, with the inflammatory waves inducing severe pain [103]. Furthermore, ionising radiation causes DNA damage which leads to repair responses, genetic mutations, or cell death, with early and late effects. Ultraviolet radiation resulting in ‘sunburn’ is one of the key inducing factors of squamous cell carcinoma and basal cell carcinoma [104]. Excessive exposure to ultraviolet radiation carries profound health risks including atrophy, pigmentary changes, skin wrinkling, and malignancy [105]. As such, radiation burn may not be a highly representative wound model for general burns treatment.

### Timeframe

The delay between establishing the skin wound in the preclinical model and applying the engineered skin product to the wound varied among the included studies, with some applying the tissue engineered product immediately post-burn, and others delaying the treatment for up to 10 days. Generally, studies involving burns patients showed that the best clinical outcomes were obtained when burns were excised and grafted at 24–48 h after the injury [106–108]. In preclinical models, treating burns outside of this optimal period using tissue engineered products may potentially reduce healing rates, although inter-species differences should also be taken into consideration when analysing the results.

There was a large variation in the endpoints of outcome evaluation used in the included studies, ranging from 7 to 90 days post-burn. Some studies also had multiple endpoints at which biopsies were taken or animals were euthanised. The normal wound healing process occurs in three phases: haemostasis and the inflammatory phase (from the time of injury to day 4–6), the proliferative phase (day 4 to 14), and finally maturation and remodelling (day 8 to 1 year or longer) [60, 109]. Studies where endpoints do not extend beyond the proliferative phase of wound healing would not allow the full effects of wound maturation and remodelling to be assessed, and therefore may not be indicative of long-term healing response. Endpoints of 1 year or longer in preclinical models would obviously be ideal, particularly for larger animals due to their longer lifespan, but this is often difficult to implement due to housing requirements and increased costs. Interestingly, despite size differences, there were no major variations in the time period required for the start of wound recovery between small and large animals, with the vast majority of included studies reporting that wound filling with granulation tissue occurred between 7 to 21 days, and only one porcine study reporting 28 days.

### Scaffolds

Biomaterials used for tissue regeneration can be generally divided into natural and synthetic biomaterials [110]. Natural biomaterials derived from biological and plant sources have been widely studied in tissue engineering because of their biocompatibility, bioactivity, and biodegradation properties, many of which possess similar structure to natural ECM. When placed in biological systems, natural biomaterials release products during biodegradation that have minimal cytotoxicity, and provide biomimetic properties to support cell adhesion and function [110]. Collagen, chitosan, and fibrin are some common examples of natural biomaterials used by studies included in this review, and are derived from protein and polysaccharide sources [111].

Collagen is a naturally occurring protein and an ubiquitous component of the skin ECM, which is commonly used as a scaffold material due to its mechanical and cell-adhesive properties [112]. Although collagen scaffolds can undergo fast biodegradation and also result in wound contraction [113], this can be mitigated by crosslinking [114]. Chitosan is a natural polymer derived from chitin. It is a popular material choice for skin regeneration scaffolds since it has good biocompatibility with skin cells, is anti-microbial, promotes wound healing, and reduces scar formation [115]. 3D chitosan nanofibrillar scaffolds produced by electrospinning showed great ability to promote skin repair both in vitro and in a mouse model [116]. However, applications of pure chitosan are often limited by low mechanical durability [117]. When combined together, collagen/chitosan scaffolds have shown the ability to promote keratinocyte migration and wound re-epithelisation in an ex vivo human skin wound model [118], as well as cutaneous wound healing in murine models when combined with BM-MSCs [119, 120]. Scaffolds with this material combination can have many advantages for skin repair such as mechanical stability, antibacterial function, and accelerated collagen synthesis through fibroblast recruitment, diluting the drawbacks of the individual materials.

Fibrin is another versatile biopolymer that can be used as a scaffold for skin regeneration. It can improve skin graft success rates, support keratinocyte and fibroblast growth [121], and convey better angiogenic properties than collagen scaffolds [112]. However, fibrin hydrogels used in skin tissue engineering also exhibit several limitations: gel shrinkage during the formation of flat sheets, low mechanical stiffness, and rapid biodegradation before the formation of vital tissue structures [121]. The usefulness of fibrin hydrogels can be extended by incorporating ECM-derived proteins to improve their biological activity, such as fibronectin, vitronectin and laminin. Injectable fibrin scaffolds that slowly release a cocktail of growth factors including PDGF, VEGF, TGF-β1, IGF, FGF, and EGF can promote skin healing through cell proliferation, collagen deposition, and tissue revascularisation [122].

Composite scaffolds can combine the advantages of several biomaterials to enhance skin regeneration, and compensate for the limitations of individual materials. One of the included studies seeded MSCs onto a biodegradable hybrid hydrogel synthesised from unsaturated arginine-based poly(ester amide) and chitosan derivative [123]. These MSC-seeded hybrid gels promoted wound closure, re-epithelialisation, granulation tissue formation, and vascularisation of third degree burns in mice. They also increased anti-inflammatory IL-10 expression and M2-like macrophages, and reduced inflammatory TNF-α expression and M1-like macrophages. While the hydrogels alone were seen to promote vascularisation, they were much more effective when seeded with MSCs. In other studies, 3D printing has been used to fabricate composite scaffolds with new material combinations for skin repair, such as a gelatine-sulfonated silk composite scaffold to overcome the deficiency of dermal vascularisation [124]. As a collagen derivative, gelatine possesses great biocompatibility and fast degradation rate, which exhibits enhanced mechanical support when combined with sulfonated silk. This 3D printed scaffold was shown to promote the regeneration of skin-like tissues in a rat skin defect model.

Another interesting but less commonly explored natural biomaterial for skin repair is the acellular amniotic membrane, obtained from the placenta following caesarean section delivery, conveying excellent biocompatibility as well as anti-inflammatory and anti-microbial effects [125]. The amniotic membrane includes amniotic mesenchymal cells (AMCs) and amniotic epithelial cells (AECs). AMCs are capable of differentiating into all three germ layers and secreting anti-inflammatory cytokines such as PGE2, IDO, HGF and TGF-β [126]. AECs have been shown to express HLA-G antigens on their surface, which are involved in the induction of immune tolerance and can effectively reduce the risk of post-transplantation rejection [126].

Synthetic polymers are also used as scaffold materials for skin regeneration as they eliminate the risk of disease transmission, and their fabrication processes can be more precisely controlled to give tailorable mechanical and chemical properties. However, most synthetic polymers lack bioactivity unless further modified [127]. An example is poly(vinyl alcohol) (PVA), which has good biocompatibility and also exhibits potential as a protein delivery system [127]. Modified PVA hydrogels were shown to be biocompatible and not elicit severe inflammatory responses for up to 12 weeks after in vivo implantation in mice. Poly (ethylene glycol) (PEG) is another commonly used synthetic polymer in skin tissue engineering [128]. PEG-based amphiphilic copolymers have versatile uses as scaffold materials, where the hydrophobic polyester components provide biodegradation and protein adhesion, while the hydrophilic PEG blocks provide better mechanical properties and elasticity [129].

### Other added substances

Other substances have been added to tissue engineered constructs involving stem cells in combination with scaffolds to improve their bioactivity and functional characteristics for skin repair in the included studies. Other cell types added in combination with stem cells can include dermal papilla cells, dermal fibroblasts, and keratinocytes [130]. Dermal papilla cells are mesenchymal cells found in the skin which regulate hair follicle growth and development [131]. Dermal fibroblasts are extensively involved in the natural wound healing process, particularly during the proliferative phase by performing collagen synthesis and contraction. Fibroblast proliferation can be induced by a variety of growth factors including PDGF, IFN-γ, IL-1, and TNF-α [109]. Keratinocytes migrate, proliferate and differentiate into the epidermis during wound healing, and also promote angiogenesis by secreting VEGF [109].

A range of bioactive substances have been added to tissue engineered constructs in the included studies, including spirulina biomass, hematoporphyrin, and the PDGF-B gene. Spirulina is a blue-green microalgae that has been shown to aid skin wound healing by promoting angiogenesis, immune cell infiltration, epithelialisation, ECM deposition, and wound contraction [132], as well as by enhancing fibroblast viability and anti-oxidative mechanisms [133]. Hematoporphyrin is a photosensitiser that can generate reactive oxygen species (ROS), providing anti-microbial effects while promoting cell proliferation, and regulating inflammatory factors and collagen remodelling [134]. PDGF-B is a growth factor that recruits pericytes essential for the stabilisation and maturation of vascular structures [135]. PDGF is produced by several cell types, including macrophages, monocytes, fibroblasts, smooth muscle cells, and endothelial cells, which can then result in chemotaxis [109]. In skin repair, PDGF-B acts as a chemical inducer that can catalyse repair cells to migrate from the wound edge to the wound bed [136], as well as stimulate the proliferation of repair cells to increase the formation of granulation tissue [137]. In addition to these, VEGF is a common pro-angiogenic factor utilised in skin repair to accelerate the early phases of wound healing by promoting neovascularisation [138]. EGF is another commonly used growth factor recognised for its therapeutic functions in stimulating skin cell growth, proliferation and differentiation [139].

## Outlook and perspectives

While stem cells have attracted significant attention in skin tissue engineering due to their ability to differentiate into tissue-specific cells and/or secrete bioactive factors to aid repair, several hurdles need to be overcome before stem cell-based products can become a standard method in clinical burns treatment. A primary hurdle in the translation of stem cell-based tissue engineered skin arises from the inherent discrepancies in skin structure and physiological response between humans and animal models [37]. For instance, murine models cannot fully replicate the pathophysiological and systemic responses to burns that humans demonstrate, which reduces the translational relevance of products tested using such models. While porcine models provide a better prediction of clinical treatment outcomes, the majority of current studies are limited in scope due to practical constraints such as housing requirements, cost, and ethics. For these reasons, the generation of in vitro skin organoids may bridge a gap between animal models and clinical response to treatment. For instance, skin organoids have been generated in 3D culture using a homogenous population of mouse pluripotent stem cells, which replicated some of the characteristics of native skin including the development of new hair follicles [140]. The same group then developed a human skin organoid, by guiding human iPSCs through a month-long process of differentiation to generate complex hair-bearing human skin tissue [141]. These skin organoids may be used to model burn wounds for testing new skin substitutes, offering short modelling times and the potential to be made patient-specific [142].

Recent developments in biofabrication technologies such as 3D bioprinting are enabling the production of anatomically-similar skin constructs, which can replicate the essential features and native functions of human skin, and be used either as a test model for new skin substitutes or developed as a therapeutic product. 3D bioprinting provides the advantages of reproducibility and customisability, allowing for accurate cell positioning and control in preparing biomimetic tissue structures [143]. Using 3D bioprinting with a medical grade bioink and mechanically extracted human skin cells, a dermis could be reconstituted in vitro, and skin cellular components could also be printed directly onto an in vivo skin wound [144]. Experiments in a murine model suggested that this technology was feasible and well-tolerated, with potential for development into a clinical treatment for patients with severe burns through a single intraoperative step. In another approach, a collagen and alginate bioink in combination with keratinocytes and fibroblasts were printed in a 3D scaffold, where cells were shown to form dense structures similar to human skin as they could migrate and proliferate on the scaffold [143]. The use of additive manufacturing in skin tissue engineering could improve standardisation and reproducibility of patient outcomes, and assist the translation of new therapeutic products into clinical applications.

The development of new biomaterials could further augment the formation of biomimetic skin constructs, potentially progressing towards full-thickness skin regeneration. For example, antibiotic-based silk fibroin (ABSF) films can accelerate burn wound healing by increasing fibroblast viability and adhesion, as well as by reducing the chance of infection at the wound site [145]. Additionally, a dextran-based hydrogel was found to stimulate neovascularisation within the first week of application when used to treat third degree burns in a pig model, resulting in rapid healing and wound closure, increased re-epithelisation and ECM remodelling, and superior reinnervation of newly formed skin tissue compared to conventional dressing treatment [146].

Future directions for using stem cells in burns treatment could involve not only the direct use of cells themselves, but also their secretory products such as extracellular vesicles (EVs), including exosomes (small EVs) and microvesicles (large EVs). The emerging role of EVs both as disease biomarkers and therapeutic agents is being increasingly recognised, as they have been identified to play key roles in intercellular communication [147, 148]. Stem cell-derived EVs have been shown to replicate the pro-regenerative functions of their parent cells, such as promoting cell proliferation and angiogenesis, and supressing apoptosis [148], pointing to their potential in being used as novel therapeutics for skin repair and burns treatment [149], possibly obviating the need to use cells and navigate associated practical hurdles. EVs from human iPSCs and MSCs are considered to have a key role in paracrine signalling, and can enhance healing in burn wounds without the associated stem cells [150]. For instance, EVs from iPSC-derived MSCs have been shown to enhance the migration of human dermal fibroblasts to stimulate vascularisation, effectively promoting wound healing by reducing scarring and improving collagen maturity [151]. In mouse model of a second degree burn, iPSC-derived EVs played a significant role in enhancing skin re-epithelisation and increasing numbers of keratinocytes [152]. MSC-derived EVs have similarly been reported to promote skin regeneration and accelerate would healing [147]. Part of the mechanisms leading to these effects may be related to the immunomodulatory properties of the parent MSCs, whereby their ability to promote anti-inflammatory M2 microphage polarisation, aid B-cell regulation, and suppress effector T-cells are replicated in the MSC-derived EVs [153, 154].

Nanotechnological approaches are now being increasingly explored to provide innovative tissue engineering solutions for treating burn wounds. Nanoparticles could be used for temporary or sustained controlled delivery of growth factors [155]. For instance, a nanofibrous skin substitute was created using electrospinning to allow programmable release of multiple angiogenic growth factors through gelatine nanoparticles [156]. This construct was designed to deliver endothelial growth factor and basic fibroblast growth factor in the early stage of wound healing to accelerate epithelisation and vascular sprouting, while PDGF and VEBF are released in the later stages to induce blood vessel maturation. Moreover, micro-/nano-robots are being increasingly explored as an exciting new field to provide targeted drug delivery that can be controlled by external sources such as magnetic forces, light, or ultrasound [157]. These could be used to deliver a range of cargo from genes and biomacromolecules to cells, and might find new applications in bioengineered skin constructs.

Nanosensors are another exciting area of development that could be integrated into tissue engineering solutions for real-time monitoring of skin repair, using biomarkers generated during the process of wound healing. For instance, novel fluorescent magnesium hydroxide nanosheets have been integrated with electrospun fibres and agarose gels to create multifunctional topical wound dressings [158]. The nanosheets provided the dressing with potent antimicrobial properties, with a strong fluorescence signature that could be used to assess the dressing degradation and functional antimicrobial capacity. Moreover, pH-responsive changes in fluorescence could act as a probe for wound acidification as an indicator of healthy wound healing. Such approaches could be considered for developing new skin wound dressings with biosensing capabilities.

Tissue engineering and regenerative medicine is rapidly evolving field. From our discussions in this review, it is evident that the use of stem cell-based tissue engineering approaches, augmented by biomaterials to assist skin repair in preclinical models of burn wounds has demonstrated promising outcomes, whereby full-thickness burns have been regenerated together with accessory structures such as hair follicles [50] in some cases. Nevertheless, further studies need to be conducted to test the safety and efficacy of these methods, and address possible variations associated with donors or treatment procedures before stem cell-based tissue engineered skin can be implemented for clinical burns treatment in human patients. This can be conducted through the optimisation of parameters, including those assessed in this review – cell types and tissue sources, cell dosage, supporting biomaterials, and treatment timeframe. Improved reporting would also help with standardisation of results from preclinical studies, particularly on quantitative data such as the thickness of newly formed skin and incidence of complications, which were rarely specified in the studies included in this review. Furthermore, as treatment responses in animal models do not directly translate to humans, it is vital that more physiologically relevant preclinical models are used to assess new therapies, or that new technologies are used to create in vitro representations of human tissue to aid proof of concept studies. By combining cross-disciplinary advances in regenerative medicine, incorporating stem cells, new biomaterials, manufacturing techniques, and nanotechnological advances, tissue engineered skin will move closer to becoming a reality for the clinical treatment of full-thickness burns.

## Supplementary Information

Below is the link to the electronic supplementary material.

Supplementary file1 (DOCX 13 KB)

## Data Availability

Not applicable.
