# Supplementary material for: Stem Cell-Based Tissue Engineering for the Treatment of Burn Wounds: A Systematic Review of Preclinical Studies
Source: Stem Cell Rev Rep. 2022 Feb 12;18(6):1926–55. doi: 10.1007/s12015-022-10341-z (PMC9391245; doi:10.1007/s12015-022-10341-z)
Supplement: Supplementary file 1 — Supplementary file1 (DOCX 13 KB) [file 12015_2022_10341_MOESM1_ESM.docx]

Search strategy in Embase:

#1 Stem cell*.mp. OR exp stem cell/

#2 burn/ OR Burn*.mp.

#3 scald.mp.

#4 #2 OR #3

#5 skin.mp. OR exp skin/

#6 dermis.mp.

#7 dermal.mp.

#8 #5 OR #6 OR #7

#9 tissue engineering/ or Tissue engineer*.mp.

#10 #9 OR #10 OR #11
#11 tissue scaffold/ OR Tissue scaffold.mp.
#12 #9 OR #10 OR #11
#13 #1 AND #4 AND #8 AND #12
#14 Limit #13 to English language
#15 Limit #14 to yr=2009-Current

Search strategy in Medline:

#1 Stem cell*.mp. OR exp Stem Cells/

#2 Burn*.mp. OR Burns/

#3 Scald.mp.

#4 #2 OR #3

#5 exp Skin/ OR Skin.mp.

#6 Dermis.mp.

#7 Dermal.mp.

#8 #5 or #6 or #7

#9 Tissue Engineering/ OR Tissue Engineer*.mp.

#10 Tissue regeneration.mp.

#11 Tissue Scaffolds/ OR Tissue Scaffold*.mp.

#12 #9 OR #10 OR #11

#13 #1 AND #4 AND #8 AND #12

#14 limit #13 to English language

#15 limit #14 to yr=”2009-Current”

Search strategy in Scopus:
#1 KEY (stem AND cell*)
#2 KEY (burn* OR scald)
#3 KEY (skin OR dermis OR dermal)
#4 TITLE-ABS-KEY (“Tissue engineer*” OR “Tissue regeneration” OR “Tissue scaffold*”)
#5 PUBYEAR > 2008
#6 LIMIT-TO (LANGUAGE, “English”)
#7 #1 AND #2 AND #3 AND #4 AND #5 AND #6

Search strategy in Web of Science:

#1 TS= (stem cell*)

#2 TS= (burn*) OR (scald)

#3 TS= (Skin) OR (dermis) OR (dermal)

#4 TS= (“tissue engineer*”) OR (“tissue regeneration”) OR (“tissue scaffold”)

#5 #1 AND #2 AND #3 AND #4
Timespan 2009-2019
